# DNA and the law in Italy: the experience of “the Perugia case”

**DOI:** 10.3389/fgene.2013.00177

**Published:** 2013-09-12

**Authors:** Carla Vecchiotti, Silvia Zoppis

**Affiliations:** Laboratory of Forensic Genetics, Section of Legal Medicine, Department of Anatomy, Histology, Forensic Medicine and Orthopaedics, University of Rome “Sapienza”Rome, Italy

**Keywords:** genetic analyses, DNA testing, forensic caseworks, reliability, interpretation

Today DNA analyses represent a method of exceptional importance for the resolution of judicial cases. On the one hand, they allow courts to secure criminal convictions, while on the other hand they can help exonerate innocent suspects. Unfortunately, DNA analyses are often considered an unbeatable and infallible method to discover the truth, with the consequence that judges feel forced either to “bow to science” or to totally refuse the genetic evidence when it is considered too complex. On the contrary, genetic investigations have limits that must always be considered and properly explained to the fact-finder by the forensic geneticist. Courts need to know what results were observed and how likely it is to observe such results under both the prosecution and defense hypotheses. This may be particularly challenging for low quantity, degraded or mixed genetic material, and is further complicated by the need to take into account the potential of (laboratory) error. Despite such circumstances, the evidence can still be informative although its probative value may be reduced.

The murder of British student Meredith Kercher in Perugia (Italy) in 2007 and the case that ensued have highlighted the limits of genetic analyses. Throughout Italy, this case has caused an intense scientific and (through the media) popular debate on the correct application of internationally recommended protocols and procedures as a preliminary quality and reliability guarantee for results presented in court. Particular attention has been drawn to the interpretation of genetic profiles derived from Low Template (LT) or Low Copy Number (LCN) DNA and mixed samples. The two defendants, Amanda Knox and Raffaele Sollecito, were convicted after the first trial but then acquitted on appeal in 2011. The Italian Supreme Court overturned the acquittal in 2013, and a new trial will be held soon.

The Appellate Court experts (author Carla Vecchiotti was one of the two experts who reviewed the case for the Court of Appeal) were asked to repeat, if possible, the genetic analyses carried out during the initial investigation on certain items and whose results led to the conviction of the two defendants: a knife, considered by the prosecution to be the murder weapon, and a bra clasp belonging to the victim. If a repetition of the analyses was impossible due to insufficient biological material, the experts were asked to examine the technical report drawn up by the scientific police in the course of the first trial. According to this document, the scientists had observed DNA profiles corresponding to the victim on the knife blade, to the defendant Amanda Knox on the knife handle and to the defendant Raffaele Sollecito on the bra clasp. The report also concluded that the correspondences between the traces and the various people involved meant that these people were the source of the DNA in question.

As for the knife, collected from the inside of a drawer in Sollecito's kitchen, the Appellate Court experts found neither traces of blood nor the presence of cellular material on the blade. The quantification analysis performed on the material collected from the blade provided a value of 5 pg/μl just in one sample, a result far below the value recommended in the technical protocols of the new generation commercial kits for STR analysis (i.e., 0.25–0.5 ng of template DNA in the PCR reaction in a maximum input volume of 17.5 μl for the PowerPlex® ESI 17 and ESX 17 System; 1 ng of template DNA in the PCR reaction in a maximum input volume of 10 μl for the AmpFlSTR® NGM SElect™ PCR Amplification Kit). Since the amount of extracted DNA would not allow the required repetition of amplification, the Appellate Court experts decided not to proceed with the genetic analyses on the swabs taken from the knife (Butler and Hill, 2010). As for the bra clasp, it was recovered and collected from the crime scene floor 46 days after the murder. It could not be analyzed by the Appellate Court experts as it had been stored by the scientific police in a tube containing extraction buffer, which made it completely rusty. Consequently, the Court experts proceeded to examine the above-mentioned technical report in order to evaluate the results obtained from the analysis of the two items.

The knife was examined first. According to the technical report, the two samples of interest were sample A, taken from the handle, and sample B, taken from the blade. Regarding the nature of the recovered material, there was no scientifically conclusive evidence to support the possible blood nature of the sample taken from the blade (sample B) in that both the generic blood test and the human species test were negative. The conclusion that exfoliated cells were present on the sample taken from the handle (sample A) was equally lacking in scientific basis. No reliable method for quantifying the DNA was employed, and the quantification performed with the Qubit Fluorimeter™ gave the result “too low” for sample B (knife blade), indicating a DNA amount below the sensitivity threshold of the Fluorimeter (200 pg/μl); therefore, presumably, a LT-DNA sample. In relation to the same sample B (knife blade), the electrophoretic graph showed peaks far below the 50 RFU threshold and allele imbalance (Hb = φa/φ b >0.60) for most of the alleles, thus indicating a LT-LCN sample. Yet, none of the recommendations issued by the international scientific community and aimed at obtaining scientifically reliable results when treating this challenging kind of samples were followed. Replicate analyses could have been performed at the time, although experts' views on how to analyze LT-DNA have been evolving since then. The main issue with that type of samples is contamination: consequently, strict protocols must be applied during the inspection, collection, and sampling of such items at the crime scene (Giardina et al., 2011). The procedures recommended to reduce laboratory contamination are equally rigorous as it is well-known that contaminant DNA at low levels may derive from reagents and other laboratory consumables, from the technical staff and from cross-contamination from sample to sample. Indeed, in the context of the Kercher murder case, transfer of a suspect's DNA into a crime scene sample was of particular importance: in fact, it appears that crime scene inspection procedures destined to minimize contamination were not carried out according to international protocols (Fischer, 2003; Laboratory Division of the Federal Bureau of Investigation, 2007; ICPO-Interpol, 2009). Furthermore, it seems that no attempts were made to discover such events.

As for the bra clasp, regarding the nature of the material recovered, there was no scientific evidence supporting the notion that flaking cells were present in the sample. The hypothesis formulated by the scientific police technical consultant about the nature of the material collected from the clasp is thus arbitrary, since it was not supported by any actual findings. After examining the electropherograms obtained from the autosomal STR analyses, the Appellate Court experts were able to assert that, for the markers D8S1179, D21S11, D19S433, D5S818, allelic peaks were interpreted in a manner that did not conform to the recommendations made in current literature/practice. In particular, peaks were considered to be stutters whose heights were above 50 RFU (D19S433), exceeded the threshold of 15% of the major allele (D8S1179, D21S11, D5S818), or were not in a stutter position (D5S818), and thus should have been considered to be alleles (Gill et al., 2006). The DNA extracted from the bra clasp thus indicates the presence of several minor contributors, which was not disclosed by the scientific police. The electropherograms obtained from Y-STRs analysis also showed (besides the peaks designated as alleles in the technical report of the scientific police) the presence of additional peaks with heights exceeding the threshold of 50 RFU (Table 1). Despite not being in a stutter position, they were not taken into consideration. Instead, the report(ing) was limited to what was in agreement with the observations on the electropherograms of the autosomal STRs. The genetic profile thus derived from a mixture of unidentified biological substances, whose larger component corresponded to the profile of the victim and whose smaller components suggest the contribution of several male sources. Defendant Raffaele Sollecito showed a profile that was compatible with the profiling results for the trace found on the bra clasp. However, considering the particular circumstances under which the item was recovered and collected, it could not be ruled out that the results obtained from the analysis of the bra clasp derived from environmental contamination and/or contamination in some phase of the collection and/or handling of the item.

**Table 1:** **Summary of the similarities and differences between the conclusions drawn by the technical consultant of the prosecution (column two) and the Appellate Court experts (column three) regarding the electropherograms obtained from the Y-STR analysis performed on the bra clasp by the scientific police**.

**LOCUS**	**Interpretation offered by the technical consultant of the prosecution**	**Interpretation offered by the Appellate Court experts**	**Alleles not reported in the scientific police technical report**
DYS456	13	13, 15	15 (↑ 82)
DYS389I	12	12, 13	13 (↑ 118)
DYS390	22	22, 23, 24	23 (↑ 76), 24 (↑ 107)
DYS389II	29	29	–
DYS458	15	15, 17	17 (↑ 63)
DYS19	14	14	–
DYS385	13,14	13, 14, 16	16 (↑ 59)
DYS393	13	12, 13, 14	12 (↑212 = 18.97% allele 13) 14 (↑ 65)
DYS391	10	10, 11	11 (↑ 183)
DYS439	11	11	–
DYS635	21	21, 22	22 (↑ 84)
DYS392	11	11	–
YGATA	11	11, 12	12 (↑ 97)
DYS437	15	14, 15	14 (↑ 144 = 18.18% allele 15)
DYS438	10	9, 10	9 (↑ 201 = 32.47% allele 10)
DYS448	20	20, 21	21 (↑ 79)

*In the brackets in column four, the symbol ↑ and the following number indicate the peak height in Relative Fluorescence Units (RFU). As for the markers DYS393, DYS437, and DYS438, the height ratio (percent) of the observed alleles is also reported*.

In conclusion, it is important to highlight some relevant issues concerning the interpretation of genetic profiles obtained from LT-LCN DNA and mixed samples. First of all, interpretation of a profile obtained for a particular item that is deciding which electrophoretic peaks are allelic and which are stutter or other artifact, must be done without reference to the suspect's profile: it is the only way to minimize the risks of bias in the interpretation of the profile derived from the evidentiary sample. Interpreting a profile derived from a sample with the suspect's reference profile at hand conflicts with the principles of scientific integrity, balance, and coherence that should underlie the practice of forensic science (Budowle et al., 2009; Thompson, 2009). It is also clear that the weight of the evidence is a fundamental issue (Gill and Buckleton, 2010), as widespread public opinion holds that if DNA found on the crime scene matches the suspect, then he must be guilty of the crime. This logically wrong understanding unfortunately also extends to a considerable number of scientists, judges, and lawyers. In fact, there is a perception that failure to convict implies a failure of science. Such a view is extremely dangerous and it is therefore important to defend the idea that whether or not a suspect is convicted is an irrelevant question for the scientist, whose responsibility must only be to correctly explain the evidence in the context of the specific case. The question of how DNA corresponding to the suspect was transferred onto an item must therefore be assessed by the judge and not by the scientist, whose role is limited to presenting the various ways in which transfer can happen and the strength of support for each of the various scenarios (Gill and Buckleton, 2010).

In Italy, the Kercher case has defined a new way of conceiving of and addressing the scientific evidence in the context of a criminal trial (Montagna, 2012): the scientific and, subsequently, legal quality of the investigations performed at the crime scene depends on the compliance with internationally standardized procedures. There is now a better awareness of the importance to follow correct crime scene procedures in order to minimize the risk of contamination and, subsequently, the loss of reliability of any results obtained. Another element that has emerged during this debate is the increased awareness, in the international scientific community, of the need to develop structured reasoning models. These should assist in the evaluation of propositions according to which the suspect is or is not one of the persons who contributed to a particular mixed biological trace, in particular in the context of LT-LCN (including additional phenomena such as drop-in, drop-out, etc.). Finally, it is worth recalling a key principle of the Italian criminal justice system, the presumption of innocence: a defendant can only be declared guilty if the prosecution proves beyond any reasonable doubt that he committed the crimes for which he is being prosecuted. If a single doubt remains, even the slightest, the defendant must be acquitted. Judges who convict in the absence of strong, unambiguous and consistent evidence violate the law (Grosso, 2011).

## References

[B1] AmpFlSTR ® NGM SElect™ PCR Amplification Kit User Guide, Applied Biosystems by Life Technologies, Carlsbad, CA, USA

[B2] Budowle B. Eisenberg A. J. van Daal A. (2009). Validity of low copy number typing and applications to forensic science. Croat. Med. J. 50, 207–217 10.3325/cmj.2009.50.207 19480017 PMC2702736

[B3] Butler J. M. Hill C. R. (2010). Scientific issues with analysis of low amounts of DNA. Profiles DNA 13. Available online at: http://www.promega.com/profiles/1301/1301_02.html

[B5] Fischer B. A. J. (2003). Techniques of Crime Scene Investigation. Boca Raton, FL: CRC Press

[B6] Giardina E. Spinella A. Novelli G. (2011). Past, present and future of Forensic DNA typing. Nanomedicine 6, 257–270 10.2217/nnm.10.160 21385128

[B7] Gill P. Brenner C. H. Buckleton J. S. Carracedo A. Krawczak M. Mayr W. R. (2006). DNA commission of the international society of forensic genetics: recommendations on the interpretation of mixtures. Forensic Sci. Int. 160, 90–101 10.1016/j.forsciint.2006.04.009 16750605

[B8] Gill P. Buckleton J. (2010). A universal strategy to interpret DNA profiles that does not require a definition of low-copy-number. Forensic Sci. Int. Genet. 4, 221–227 10.1016/j.fsigen.2009.09.008 20457049

[B4] Grosso C. F. (2011). L'incerta Prova Scientifica. Torino: La Stampa

[B9] ICPO-Interpol. (2009). Handbook on DNA Data Exchange and Practice- Recommendations from the Interpol DNA Monitoring Expert Group, 2nd Edn. Lyon: OIPC-INTERPOL

[B10] Laboratory Division of the Federal Bureau of Investigation. (2007). Handbook of Forensic Services, 6th Edn. Quantico, VA: Kim Waggoner

[B11] Montagna M. (2012). L'assassinio di Meredith Kercher - Anatomia del Processo di Perugia. Collana: Oltre ogni ragionevole dubbio

[B12] PowerPlex® ESI 17 and ESX 17 System Technical Manual, Promega Corporation, Madison, WI

[B13] Thompson W. C. (2009). Painting the target around the matching profile: the Texas sharpshooter fallacy in forensic DNA interpretation. Law Prob. Risk 8, 257–276 10.1093/lpr/mgp013

